# Abbreviated Point-of-Care-Ultrasound Volume Assessment for Outpatient Hemodialysis Patients

**DOI:** 10.1016/j.mayocpiqo.2026.100766

**Published:** 2026-10-09

**Authors:** Leanna V. Ritchie, Michael G. Heckman, Zhihui Fang, Lyle W. Baker, Fouad T. Chebib, LaTonya J. Hickson, Sandhya Manohar, Ivan E. Porter, Nabeel Aslam, Christopher L. Trautman, Michael A. Mao

**Affiliations:** a Division of Nephrology and Hypertension, Mayo Clinic, Jacksonville, FL; b Division of Clinical Trial and Biostatistics (M.G.H., Z.F.), Mayo Clinic, Jacksonville, FL

## Abstract

**Objective:**

To evaluate the safety and feasibility of an abbreviated lung and inferior vena cava (IVC) point-of-care ultrasound (POCUS) protocol for outpatient hemodialysis volume assessment when compared with standard practice.

**Patients and Methods:**

In this prospective case-control study, adults receiving long-term in-center hemodialysis underwent abbreviated POCUS-assisted assessment (n=15, 34%) or standard clinical assessment (non-POCUS, n=29, 66%). POCUS was performed during dialysis using a 6-zone lung ultrasound protocol with IVC assessment. Hypervolemia was defined as ≥3 B lines in ≥2 lung zones with bilateral involvement or an IVC diameter ≥2.1 cm with <50% collapsibility. The primary outcome was change in estimated dry weight (EDW) guided by POCUS when compared with standard clinical assessment. Secondary outcomes included falls, all-cause and hypervolemia-related hospitalizations, antihypertensive medication changes, and mortality.

**Results:**

All POCUS participants (15/15, 100%) met hypervolemia criteria at the initial assessment and underwent EDW reduction; 46.7% met criteria at repeat assessment. Median POCUS examination time was 6 minutes (range 4-10). Reductions in prescribed EDW occurred more frequently in the POCUS group (100% vs 41.4%). Postdialysis weight remained higher relative to new prescribed EDW in the POCUS group at study end. No significant differences were observed in intradialytic blood pressure (*P*=.24), falls (*P*=.98), or hospitalizations (*P*=.67), and no hypervolemia-related hospitalizations (*P*=.54) occurred in the POCUS group.

**Conclusion:**

An abbreviated 6-zone lung and IVC POCUS protocol is feasible and safe in outpatient hemodialysis and prompts earlier EDW adjustment. Larger randomized studies are needed to determine its impact on sustained volume control and clinical outcomes.

Mobile health technologies (MHT) have transformed the landscape of health care practice. MHTs are increasingly portable, cost-effective, and user-friendly. The point-of-care ultrasound (POCUS) is a portable MHT ultrasound probe that connects to a mobile phone to allow timely and rapid bedside ultrasound assessment to guide early diagnostic decision-making. These POCUS tools have been shown to have superior accuracy and earlier detection of hypervolemia compared with the health care practitioner’s traditional physical examination.^1^ However, this tool is not routinely used as standard practice for volume assessment in most outpatient dialysis populations.

End-stage kidney disease (ESKD) patients requiring dialysis have considerably increased morbidity, mortality, and health care expenditure compared with the general population.^2^ Hypervolemia in the ESKD dialysis population is a frequent challenge that is directly associated with increased morbidity, mortality, hospitalizations, and an inverse correlation with quality of life.^3^^,^^4^ The clinical determination of hypervolemia remains a persistent barrier to timely management and prevention of its associated poor patient outcomes. Symptoms and signs of heart failure have been shown as poor predictors for death by cardiac event, and physical exam findings have limited sensitivity and specificity.^4^ Other laboratory markers of volume overload, such as brain natriuretic peptide (BNP) and N-terminal pro-BNP, can be elevated in kidney disease, require blood sampling, have delayed results, and have limited and debated utility for volume assessment.^5^, ^6^, ^7^

POCUS implementation in this vulnerable population can provide a safe, rapid diagnostic tool to allow for earlier identification of hypervolemia to mitigate patient harm and complications. ESKD patients requiring dialysis may uniquely benefit from MHT POCUS because timely, accurate volume assessment can guide therapeutic interventions that can be rapidly implemented through dialysis. Lung ultrasound as part of POCUS is a validated tool for pulmonary vascular congestion in patients with heart failure and cardiovascular disease. In 2016, the lung water by ultrasound-guided treatment (LUST) study compared with POCUS assessment of hypervolemia in ESKD patients to traditional clinical physical examination. Lung ultrasound identified hypervolemia in 65% of patients, compared with physical examination findings of pulmonary rales in only 21% and pedal edema in 10%.^8^ Subsequently, a randomized control trial (RCT) in 2020 utilized POCUS-guided volume assessment in ESKD patients to facilitate earlier detection of hypervolemia.^9^ Notably 79% of patients were found to have clinically relevant POCUS findings that changed their treatment plan. These findings were further supported by a RCT that reported persistent volume relief in 78% of patients assessed with POCUS in ESKD patients compared with traditional physical examination for volume assessment.^10^ Current evidence thus supports POCUS to improve diagnostic accuracy and reduce time to therapeutic intervention, although POCUS techniques and effects on downstream clinical outcomes remain variable.

The LUST study^8^^,^^11^ used the pre-established 28-zone protocol (16 in right hemithorax and 12 in left hemithorax) for focused lung POCUS assessment. This protocol was first described by Jambrik et al^11^ in 2004 and subsequently used as the lung ultrasound protocol in several studies.^7^^,^^10^ However, routine use of the 28-zone protocol POCUS examination may be challenging in busy dialysis units because of patient positioning in dialysis recliners and provider time constraints. Efforts to simplify the 28-zone protocol have been previously made by Raz et al, who compared and found similar improved detection of hypervolemia with an alternative 16-zone, 12-zone, and 8-zone protocols when compared with physical examination.^12^ Subsequently, Danish et al^13^ reported that the 6-zone lung ultrasound, adapted from the bedside lung ultrasonography in emergency protocol due to its ease and time efficiency, had comparable diagnostic performance with chest or computed tomography imaging studies in intensive care unit patients.^13^^,^^14^ Our study aimed to further extend the utility of an abbreviated POCUS examination for in-center dialysis patients.

Our prospective case-control study evaluates the feasibility and real-world effectiveness of employing an abbreviated POCUS examination to assess volume status and adjust ultrafiltration treatment goals in ESKD patients undergoing maintenance in-center hemodialysis at the Mayo Clinic Florida dialysis unit, compared with standard clinical practice. We used the modified, more concise, and time-efficient 6-zone POCUS protocol to report safety, feasibility, and efficacy to affect clinical outcomes. The primary clinical outcome was the change in estimated dry weight (EDW). Secondary outcomes included adverse symptoms, intradialytic hypotension, falls, hospitalizations, antihypertensive medication changes, and all-cause mortality.

## Patients and Methods

### Study Subjects

ESKD patients on long-term in-center hemodialysis at the Mayo Clinic outpatient dialysis unit in Jacksonville, Florida were included in this study. Patients were eligible for study participation if they were (1) adults aged ≥18 years, (2) diagnosed with ESKD, (3) receiving long-term outpatient hemodialysis therapy for ≥3 months, (4) receiving hemodialysis treatment lasting ≥3 hours, (5) compliant with treatment (no missed dialysis treatments or shortened treatments), and (6) able to provide informed consent. Language interpreters and consent forms in other languages were available on request. Exclusion criteria included (1) patients receiving short-term hemodialysis, (2) noncompliance, defined as missing more than 2 hemodialysis treatments in the 4-week period before study enrollment, (3) previous solid organ transplant, and (4) hemodialysis treatments lasting <3 hours. This study was reviewed and approved by the institutional review board and participants have signed written consent.

A total of 44 patients who underwent long-term in-center hemodialysis at the Mayo Clinic in Jacksonville, Florida between August 1, 2023 and April 30, 2024 were included in this study. Of the 44 patients, a subset of 15 (34%) patients were randomly selected as the interventional (POCUS) group from the Monday-Wednesday-Friday and Tuesday-Thursday-Saturday hemodialysis shifts. These were randomly selected after patient and provider consent and availability to perform the study. The remaining 29 (66%) patients from the same dialysis shifts and study period were selected as the control (non-POCUS) group using the same inclusion and exclusion criteria noted above. Because of the nature of intervention and assessment, the intervention assignment was not blinded.

### Volume Assessment

In the control group, volume status was assessed according to standard clinical practice. Standard practice includes review of the available vital signs, volume and weight trends, laboratory data, and history and physical examination, including, but not limited to, lung auscultation, abdominal examination, and assessment of extremities for edema.

In the POCUS group, volume status was assessed by physical examination and an abbreviated POCUS exam using the Butterfly IQ+ full body device ultrasound probe.^15^ A study investigator was trained on POCUS lung ultrasound utilizing the Mayo Clinic simulation center education program consisting of a total of ∼120 minutes. A focused POCUS evaluation for volume status was performed by study investigators during the last 60 minutes of a regularly scheduled hemodialysis session. Assessment was performed in the last 60 minutes to capture when patients may be closest to their euvolemic state while minimizing prolonging patient’s time at dialysis, patient burden, and workflow interruptions. For the POCUS examination, each participant lay in the supine position at a 30-degree head of elevation for lung and inferior vena cava (IVC) ultrasound. Patient privacy was maintained without disruption to the dialysis treatment through the use of medical privacy curtains. The examination was performed with a curvilinear probe on the device’s lung setting at the mid-clavicular, mid-axillary, and posterior axillary lines bilaterally (6-zone protocol) and the IVC (Supplemental Figure 1, available online at http://www.mcpiqojournal.org).^16^ Specifically, the 6-zone lung ultrasound protocol was labeled as L1, L2, L3, R1, R2, and R3. ‘L’ indicates the left lung and ‘R’ indicates the right lung. Zone 1 correlated with the 3rd or 4th intercostal space at the mid-clavicular line to evaluate the upper lung lobe. Zone 2 correlated with the 5th or 6th intercostal space at the anterior axillary line to evaluate the middle lobe or middle lung. Zone 3 correlated with the 9th or 10th intercostal space at the posterior axillary line to evaluate the lower lung lobe (Supplemental Figure 1). Lung congestion was categorized as ≥3 ultrasound B lines in ≥2 lung zones with bilateral involvement. The IVC was assessed using the abdominal setting of the curvilinear probe in the transverse view. Longitudinal view was only used for confirmation of transverse findings in some patients.

A traditional clinical physical examination was also performed immediately before the focused POCUS examination by the study investigator to allow for outcome comparison. Lung auscultation was performed in the anterior superior and posterior basilar sites with patients in a supine or upright position, respectively. Patients were instructed to take slow full inhalations and exhalations during assessment. Pulmonary rales were assessed as absent (0) or present (1). For peripheral edema of the abdomen or lower extremities, the following scale was used: 0, no pitting edema; 1, edema with persistent thumb depression <5 seconds; 2, persistent thumb depression 5-10 seconds; and 3, persistent thumb depression >10 seconds. To meet physical examination criteria for hypervolemia, patients had to have evidence of ≥1+ pitting edema or the existence of rales on lung auscultation.

### Intervention Through EDW Adjustment

At the Mayo Clinic Florida outpatient dialysis clinic, hypervolemia is typically treated by a standard protocol of 0.5-1.0 kg adjustments to the targeted EDW based on clinical volume assessment. In our control group, the EDW was adjusted per standard practice based on their nephrology provider’s clinical assessment. In the POCUS group, we opted for a conservative protocol of 0.5 kg EDW reductions if patients met POCUS criteria for hypervolemia to maximize safety.

POCUS hypervolemia criteria consisted of an IVC diameter of ≥2.1 cm with <50% diametric collapsibility, or the detection of ≥3 confluent ultrasound B-lines in ≥2 of 6 lung zones with at least 1 zone positive bilaterally. The IVC collapsibility was defined as a reduction to <50% of the maximal IVC diameter during a sniff maneuver. Changes to decrease EDW per POCUS assessment were made no sooner than every 2 weeks (maximum of 1.0 kg over the study period).

### Study Phases

The study period was divided into 4 phases including a pre-study (14 days), weeks 1-3 (14 days), weeks 3-5 (14 days), and a 3 months post-study (90 days), (Figure 1). Data collection occurred during each phase as specified. During weeks 1-3 and weeks 3-5 phases, one intervention change to EDW during each phase was performed if patients met POCUS hypervolemic criteria. In the 14-day pre-study and 3-month post-study phases, no study-related interventions were made, and the patient’s hemodialysis prescriptions, including targeted EDW, were managed by standard clinical practice. The pre-study phase was used to collect baseline data on volume management.

**Figure 1:**
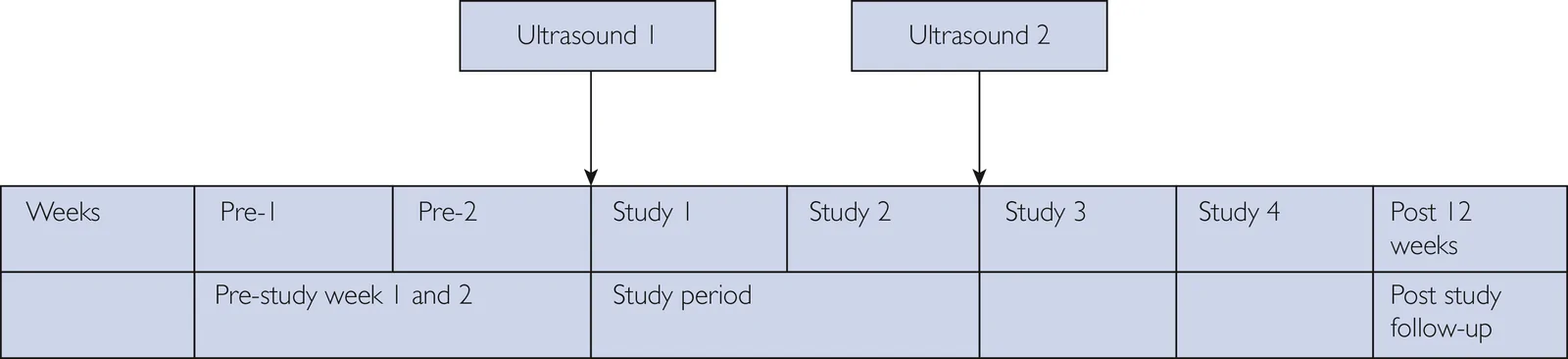
Timeline of study intervention. Two weeks of pre-study observation are noted as Pre-1 and Pre-2. Study period encompasses 4 weeks composed of Study 1 through 4. Initial POCUS with potential EDW challenge occurs in Study 1. Second POCUS with potential EDW challenge occurs in Study 3. Study completion at end of week 4. Follow-up occurred until post-12 weeks of study initiation. EDW, estimated dry weight; POCUS, point-of-care ultrasound.

### Outcomes

The primary outcome was change in EDW guided by POCUS assessment of hypervolemia compared to traditional assessment. Secondary outcomes included the incidence of falls, all-cause hospitalizations, hospitalizations due to hypervolemia, antihypertensive medication reduction, and volume status. In the POCUS group, additional patient-reported experience and safety outcomes were assessed as a result of their intervention. Occurrence of these binary outcomes at any time over the entire 4-month study period for each patient was recorded.

### Data Collection

Information was collected regarding baseline patient characteristics (age, sex, and body mass index), race, ethnicity, dialysis vintage (time in months since dialysis initiation), hypertension, diabetes mellitus, diastolic dysfunction, heart failure, left ventricular ejection fraction, coronary artery disease, atrial fibrillation, tobacco use, lung rales, leg/abdomen edema, meeting EDW before study, length of dialysis treatment (minutes), dialysis access type, POCUS information (ultrasound B line number per zones: R1, R2, R3, L1, L2, L3, time for ultrasound exam, IVC diameter, and IVC collapsibility) and study outcomes, including euvolemia status, challenge EDW, meeting EDW goal, weights, blood pressures (BP), any hospitalization, hospitalization for hypervolemia, antihypertensive (BP) medication reduction, falls, serum albumin, and serum hemoglobin.

Systolic and diastolic BP at the beginning (pre-HD) and end (post-HD) of each dialysis treatment, and the lowest intradialytic BP, were recorded for each hemodialysis treatment. These systolic and diastolic BPs were averaged over 6 treatments during their corresponding study phase. Inter-dialytic weight gain was assessed by pre-hemodialysis and post-hemodialysis weights, which were averaged over six treatments during the corresponding study phase.

Patient experience, adverse symptoms (lightheadedness, dizziness, muscle cramping, nausea, weakness, shortness of breath, or fatigue), and the need for intervention during dialysis (for cramping or hypotension) per standard dialysis protocol were assessed at the beginning of the study and at week 4. Patients were asked to rate the frequency of their symptoms on a scale of 0 (less than 1 day per week; mild), 1 (less than half of the days per week; moderate), or 2 (more than half of the days per week; severe) (see Supplemental Figure 2, available online at http://www.mcpiqojournal.org).

### Statistical Analyses

Continuous variables were summarized using the sample median and range. Categorical variables were summarized as absolute counts and percentages. Comparisons of baseline characteristics between POCUS and non-POCUS patients were made using the Wilcoxon rank sum test (continuous variables) or Fisher’s exact test (categorical variables). Outcomes were compared between the POCUS and non-POCUS groups using both unadjusted and multivariable linear regression models (continuous outcomes) and logistic regression models (binary categorical outcomes). For linear regression models, mean differences and 95% CIs were estimated and are interpreted as the difference in means of the given outcome between POCUS and non-POCUS patients (i.e., POCUS minus non-POCUS). For logistic regression models, odds ratios (ORs) and 95% CIs were estimated and are interpreted as the multiplicative increase in the odds of the given outcome for POCUS patients compared to non-POCUS patients. Multivariable models were adjusted for all baseline characteristics that differed between POCUS and non-POCUS patients with a *P*<.10, taking care to avoid adjusting for more variables than the sample size allows for per recommended guidelines.^17^ No adjustment for multiple testing was made in these exploratory analyses, and *P*<.05 were considered statistically significant. All statistical tests were 2-sided. Statistical analyses were performed using SAS (version 9.4; SAS Institute, Inc).

## Results

### Baseline Characteristics

This study included 44 in-center hemodialysis patients with a median age of 68.7 years (POCUS) and 67.2 years (non-POCUS). No statistically significant differences were identified between the 2 study groups (all *P*≥.05). In the POCUS group, 100% (n=15) of patients had hypertension, 60% (n=9) had diabetes mellitus, 53% (n=8) had heart failure and cardiovascular disease, 60% (n=9) had former or current tobacco use, and 20% (n=3) had atrial fibrillation. Although not statistically significant, the POCUS group had a higher prevalence of heart failure (53.3% POCUS compared with 37.9% non-POCUS). The POCUS group also reported trends toward longer dialysis vintage (median 31 vs 26 months, *P*=.076) and a higher prevalence of leg or abdominal edema (46.7% vs 20.7%, *P*=.092), compared with the non-POCUS group. Before study initiation, there was no statistical difference between groups for the number of patients meeting goal EDW with hemodialysis volume removal. However, comparatively, fewer POCUS patients met their prescribed EDW before the study (60% vs 72.4%, *P*=.50). A comparison of baseline characteristics between POCUS and non-POCUS patients is provided in Table 1.

**Table 1:** Comparison of Baseline Characteristics Between POCUS and Non-POCUS Long-Term In-Center Hemodialysis Patients^a^^,^^b^

Variable	POCUS patients (n=15)		Non-POCUS patients (n=29)		*P*
	N	Median (minimum, maximum) or no. (%) of patients	N	Median (minimum, maximum) or no. (%) of patients	
Age (y)	15	68.7 (38.7-92.8)	29	67.2 (23.8-86.2)	.38
Sex (male)	15	9 (60.0%)	29	19 (65.5%)	.75
BMI (kg/m^2^)	15	27.7 (18.9-40.8)	29	23.4 (16.8-35.9)	.34
Race	15		29		.44
Caucasian		11 (73.3%)		16 (55.2%)	
African American		3 (20.0%)		7 (24.1%)	
Asian		1 (6.7%)		6 (20.7%)	
Ethnicity (Hispanic or Latino)	15	1 (6.7%)	29	0 (0.0%)	.34
Dialysis vintage (mo)	15	31 (8-88)	29	26 (1-155)	.076
Hypertension	15	15 (100.0%)	29	27 (93.1%)	.54
Diabetes mellitus	15	9 (60.0%)	29	11 (37.9%)	.21
Diastolic dysfunction	15	6 (40.0%)	29	9 (31.0%)	.74
Heart failure	15		29		.23
No		7 (46.7%)		18 (62.1%)	
Reduced ejection fraction		2 (13.3%)		1 (3.4%)	
Preserved ejection fraction		6 (40.0%)		7 (24.1%)	
Combined heart failure		0 (0.0%)		3 (10.3%)	
Left ventricular ejection fraction (%)	14	65 (23-76)	26	62 (37-79)	.89
Coronary artery disease	15	8 (53.3%)	29	15 (51.7%)	>.99
Atrial fibrillation	15	3 (20.0%)	29	9 (31.0%)	.50
Tobacco use	15	9 (60.0%)	29	11 (37.9%)	.21
Lung rales	15	0 (0.0%)	29	0 (0.0%)	>.99
Leg/abdomen edema	15	7 (46.7%)	29	6 (20.7%)	.092
Meeting EDW before study	15	9 (60.0%)	29	21 (72.4%)	.50
Length of dialysis treatment (minutes)	15	225 (180-255)	29	210 (180-270)	.74
Dialysis access type	15		29		.90
AV fistula		10 (66.7%)		18 (62.1%)	
AV graft		2 (13.3%)		3 (10.3%)	
Tunneled catheter		3 (20.0%)		8 (27.6%)	
Average pre-HD weight pre-study	15	85.2 (47.3-130.6)	29	74.9 (38.9-124.1)	.29
Average post-HD weight pre-study	15	83.5 (46.1-127.7)	29	72.9 (37.9-123.8)	.42
Serum albumin	15	3.9 (2.9-4.5)	29	3.9 (3.1-4.5)	>.99
Serum hemoglobin	15	10.1 (8.3-12.1)	29	10.6 (7.6-12.3)	.20

a EDW, estimated dry weight.

b *P* values result from a Wilcoxon rank sum test (continuous variables) or Fisher’s exact test (categorical variables).

### POCUS Assessment

POCUS assessment results are displayed in Figure 2. The variable output indicates the number of observed B lines per each of the 6 zones in the protocol. There was variability in distribution of B lines between each zone without an observable relevant pattern between zones. The median time for ultrasound examination was 6 minutes with a range of 4-10 minutes. The average POCUS assessment time decreased from 7.2 minutes to 6.1 minutes from first to second assessment. The median IVC diameter was 1.6 cm with a range of 1.3 to 2.0 cm.

**Figure 2:**
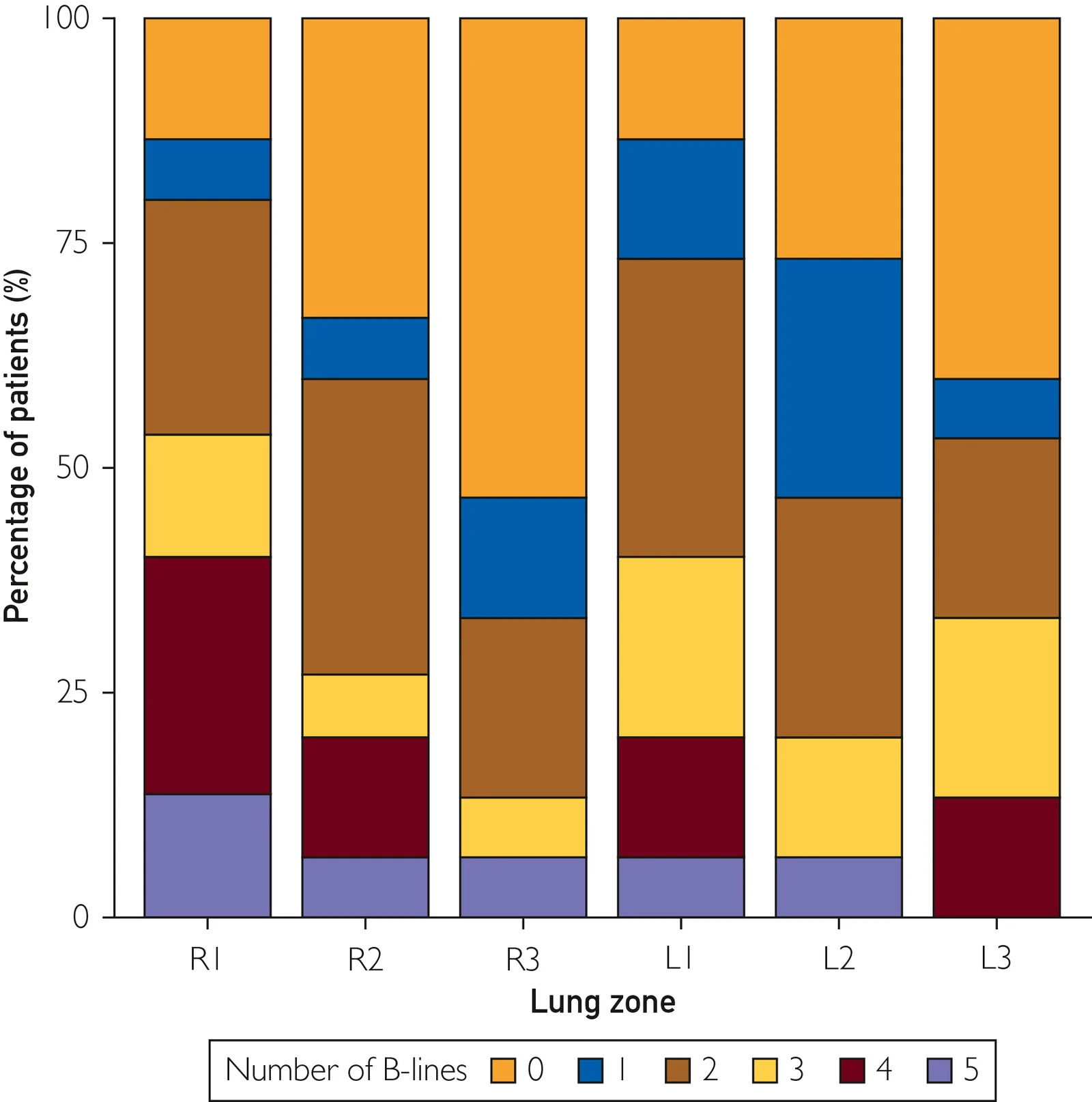
POCUS lung assessment results. Frequency distribution of ultrasound B-lines across the six predefined lung zones during the initial POCUS assessment in patients receiving outpatient in-center hemodialysis. POCUS, point-of-care ultrasound.

During the first POCUS assessment, all 15 (100%) of POCUS patients met study criteria for hypervolemia by B-line criteria and thus had their EDW challenged by a decrease of 0.5 kg. During the second POCUS assessment at week 3 of the study period, only 46.7% (n=7) of patients met hypervolemic study criteria by B-line criteria and had their EDW challenged (Supplemental Figure 3, available online at http://www.mcpiqojournal.org). Comparatively, the median IVC diameter was 1.6 cm and below the cutoff for hypervolemia of 2 cm. 60% of patients reported <50% collapsibility of the IVC. Therefore, 40% more patients in our cohort met criteria for hypervolemia utilizing ultrasound B-lines criteria rather than IVC distention or size.

### Comparison of Study Groups With and Without POCUS

In unadjusted analysis, more POCUS patients had a change in prescribed EDW target compared with non-POCUS patients (100.0% vs 41.4%, *P*<.001). However, the absolute changes in weight remained positive in POCUS compared with non-POCUS patients, as reflected by the difference between end of study weight and new EDW (mean difference: 2.91, *P*<.001, Figure 3) and the difference between end of study and old EDW (mean difference: 2.49, *P*=.005). This indicates that more of the non-POCUS group successfully achieved their prescribed EDW when compared with the POCUS group, appreciating that fewer non-POCUS patients had prescribed lowering of their EDW target. Outcomes are compared between POCUS and non-POCUS patients in Table 2.

**Figure 3:**
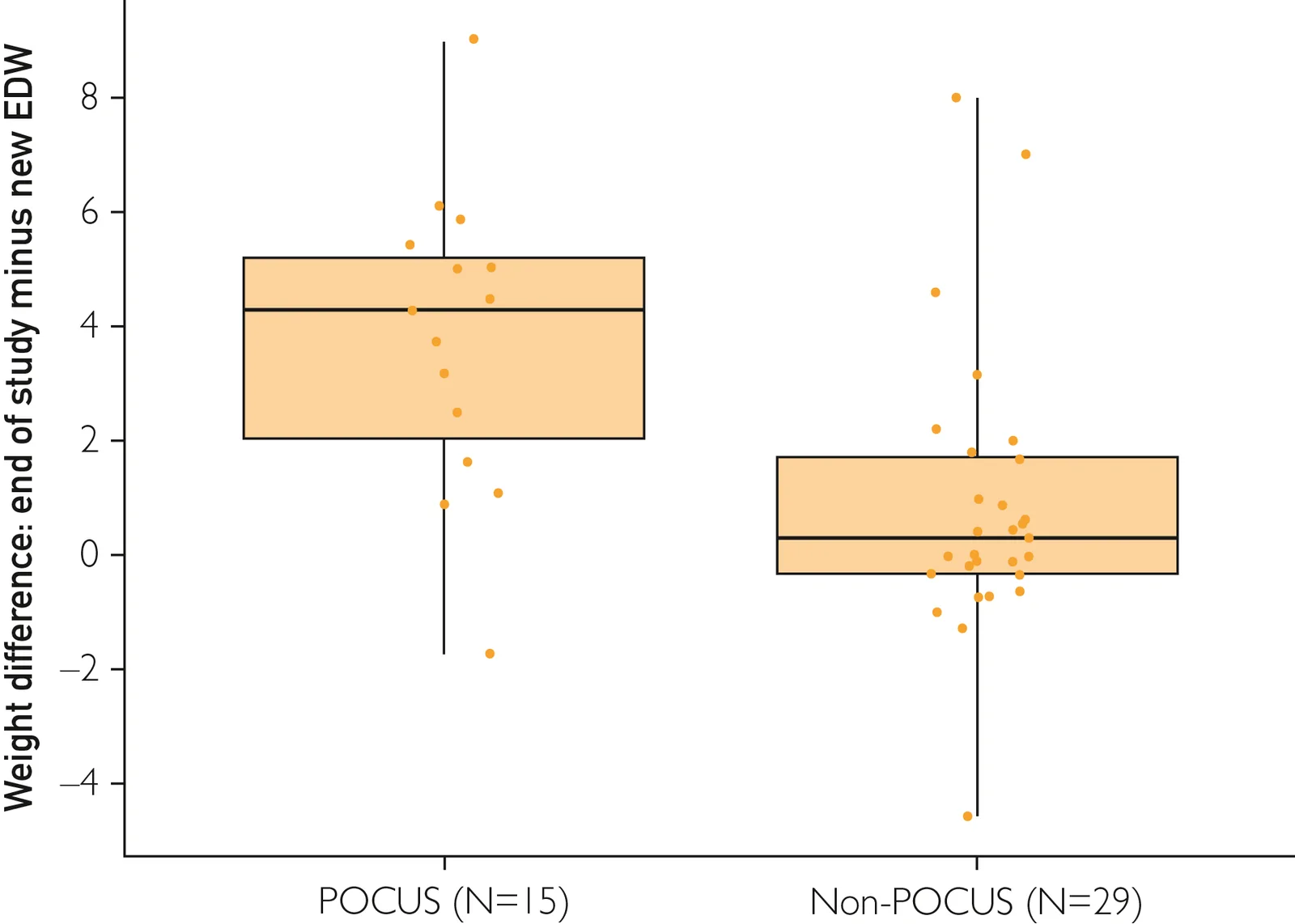
End of study period weight minus new EDW in POCUS and non-POCUS patients. Boxplots show the difference between end of study weight and newly prescribed EDW in the POCUS and non-POCUS groups. The difference was significantly greater in POCUS patients than in non-POCUS patients (mean difference, 2.91 kg; *P*<.001). EDW, estimated dry weight; POCUS, point-of-care ultrasound.

**Table 2:** Comparisons of Outcomes Between POCUS and Non-POCUS Patients^a^^,^^b^

Outcome	POCUS patients (n=15)		Non-POCUS patients (n=29)		Association Measure	Difference between POCUS patients and Non-POCUS patients (reference group)			
						Unadjusted analysis		Multivariable analysis	
	N	Median (Minimum, Maximum) or No. (%) of patients	N	Median (Minimum, Maximum) or No. (%) of patients		Estimate (95% CI)	*P*	Estimate (95% CI)	*P*
Euvolemia	15	8 (53.3%)	29	23 (79.3%)	Odds ratio	0.30 (0.08-1.16)	.08	0.29 (0.08-1.16)	.18
Challenge EDW	15	15 (100.0%)	29	12 (41.4%)	Odds ratio	N/A^c^	<.001	N/A^1^	N/A^c^
Any hospitalization	15	5 (33.3%)	29	12 (41.4%)	Odds ratio	0.71 (0.19-2.61)	.6	0.75 (0.20-2.81)	.67
Hospitalization for hypervolemia	15	0 (0.0%)	29	2 (6.9%)	Odds ratio	N/A^c^	.54	N/A^1^	N/A^c^
Blood pressure medication reduction	15	1 (6.7%)	29	3 (10.3%)	Odds ratio	0.62 (0.06-6.52)	.69	N/A^d^	N/A^d^
Falls	15	1 (6.7%)	28	2 (6.9%)	Odds ratio	0.96 (0.08-11.58)	.98	N/A^d^	N/A^d^
Serum albumin	15	3.9 (2.9-4.5)	29	3.9 (3.1-4.5)	Mean difference	−0.01 (−0.24 to 0.21)	.89	0.01 (−0.23 to 0.25)	.93
Serum hemoglobin	15	10.1 (8.3-12.1)	29	10.6 (7.6-12.3)	Mean difference	−0.42 (−1.09 to 0.25)	.21	−0.27 (−0.92 to 0.39)	.41
Systolic blood pressure									
Average low wks 1-3	15	114.3 (89.3-136.2)	29	109.0 (82.0-145.0)	Mean difference	0.37 (−9.52 to 10.26)	.94	−0.77 (−10.19 to 8.64)	.87
Average low wks 3-5	15	104.7 (94.2-139.8)	29	113.0 (75.0-157.0)	Mean difference	−5.12 (−16.40 to 6.16)	.36	−7.16 (−17.93 to 3.60)	.19
Diastolic blood pressure									
Average low wks 1-3	15	65.2 (52.5-94.0)	29	68.0 (44.0-100.0)	Mean difference	−1.09 (−8.76 to 6.58)	.77	−1.34 (−8.74 to 6.06)	.72
Average low wks 3-5	15	65.2 (49.0-87.1)	29	71.0 (52.0-114.0)	Mean difference	−3.25 (−11.63 to 5.14)	.44	−4.48 (−12.56 to 3.60)	.27
Lowest average blood pressure differences (first value listed minus second value listed)									
Pre-study and wk 3-5 lowest average intradialytic systolic	15	1.7 (−19.8 to 31.0)	29	−2.0 (−39.0 to 25.0)	Mean difference	4.83 (−4.67 to 14.32)	.31	5.98 (−4.08 to 16.03)	.24
Pre-study and wk 3-5 lowest average intradialytic diastolic	15	−0.3 (−14.0 to 12.9)	29	−1.0 (−25.0 to 15.0)	Mean difference	0.63 (−4.43 to 5.70)	.8	0.86 (−4.56 to 6.28)	.75
Pre-study and wk 1-3 lowest average intradialytic systolic	15	0.0 (−32.0 to 27.0)	29	0.0 (−21.0 to 21.0)	Mean difference	−0.67 (−8.29 to 6.95)	.86	−0.41 (−8.55 to 7.73)	.92
Pre-study and wk 1-3 lowest average intradialytic diastolic	15	0.0 (−29.2 to 8.1)	29	2.0 (−23.0 to 18.0)	Mean difference	−1.52 (−7.27 to 4.23)	.6	−2.28 (−8.39 to 3.83)	.46
Pre and post blood pressure differences (first value listed minus second value listed)									
Pre-dialysis systolic BP pre-study and wks 3-5	15	−4.0 (−22.0 to 15.0)	29	−6.0 (−21.0 to 17.0)	Mean difference	0.45 (−6.76 to 7.65)	.90	1.34 (−6.29 to 8.97)	.72
Pre-dialysis diastolic BP pre-study and BP wks 3-5	15	−2.0 (−14.0 to 9.0)	29	−5.0 (−17.0 to 14.0)	Mean difference	2.08 (−2.70 to 6.87)	.39	2.35 (−2.76 to 7.46)	.36
Postdialysis systolic BP pre-study and wks 3-5	15	2.0 (−18.0 to 22.0)	29	−6.0 (−42.0 to 12.0)	Mean difference	6.00 (−2.19 to 14.20)	.15	4.34 (−4.24 to 12.93)	.31
Postdialysis diastolic BP pre-study and wks 3-5	15	1.0 (−11.0 to 13.0)	29	−1.0 (−34.0 to 26.0)	Mean difference	2.30 (−4.41 to 9.00)	.49	2.20 (−4.85 to 9.24)	.53
Pre-dialysis and postdialysis systolic BP wks 3-5	15	13.0 (−6.0 to 39.0)	29	7.0 (−27.0 to 44.0)	Mean difference	7.38 (−2.42 to 17.19)	.14	5.44 (−4.49 to 15.37)	.28
Pre-dialysis and postdialysis diastolic BP wks 3-5	15	6.0 (−6.0 to 22.0)	29	4.0 (−19.0 to 18.0)	Mean difference	1.60 (−3.89 to 7.08)	.56	1.35 (−4.48 to 7.17)	.64
Pre-dialysis and postdialysis systolic BP wks pre-study	15	9.0 (−12.0 to 34.0)	29	11.0 (−21.0 to 32.0)	Mean difference	1.83 (−7.47 to 11.12)	.69	2.43 (−6.91 to 11.78)	.60
Pre-dialysis and postdialysis diastolic BP wks pre-study	15	6.0 (−14.0 to 15.0)	29	2.0 (−21.0 to 26.0)	Mean difference	1.38 (−5.01 to 7.76)	.67	1.50 (−4.84 to 7.84)	.64
Weight (kg)									
Meeting EDW goal wks 3-5	15	5 (33.0%)	29	17 (58.6%)	Odds ratio	0.35 (0.10-1.30)	.12	0.24 (0.05-1.06)	.059
Average pre-HD wks 1-3	15	86.4 (47.3-30.0)	29	76.2 (40.1-125.3)	Mean difference	5.70 (−8.70 to 20.09)	.43	−1.75 (−14.72 to 11.22)	.79
Average pre-HD wks 3-5	15	86.5 (48.4-130.8)	29	75.1 (43.3-125.7)	Mean difference	6.31 (−8.19 to 20.81)	.38	−1.13 (−14.23 to 11.97)	.86
Average 3 mo post-study	15	84.6 (48.1-129.9)	29	75.6 (42.7-124.2)	Mean difference	5.58 (−8.77 to 19.93)	.44	−1.50 (−14.68 to 11.69)	.82
Post-HD weight 3 mo post-study	15	80.2 (46.9-127.0)	29	73.7 (42.9-121.0)	Mean difference	5.26 (−8.48 to 19.01)	.44	−1.25 (−14.08 to 11.59)	.85
Average post-HD weight wks 3-5	15	83.7 (47.7-127.5)	29	73.2 (42.4-125.2)	Mean difference	5.23 (−8.83 to 19.3)	.46	−1.90 (−14.65 to 10.85)	.76
Old EDW	15	81.0 (45.0-128.0)	29	72.0 (37.5-120.0)	Mean difference	5.43 (−8.09 to 18.95)	.42	−1.74 (−13.81 to 10.33)	.77
New EDW	15	80.5 (45.0-127.5)	29	71.0 (39.0-120.0)	Mean difference	5.01 (−8.63 to 18.65)	.46	−2.18 (−14.40 to 10.04)	.72
End of study	15	84.4 (48.2-130.0)	29	73.3 (39.9-127.0)	Mean difference	7.93 (−6.38 to 22.23)	.27	0.73 (−12.32 to 13.78)	.91
Weight differences (kg, first value listed minus second value listed)									
End of study and new EDW	15	4.3 (−1.7 to 9.0)	29	0.3 (−4.6 to 8.0)	Mean difference	2.91 (1.31-4.52)	<.001	2.91 (1.19-4.62)	.001
Post-HD at 3 months and new EDW	15	1.9 (−10.0 to 6.3)	29	1.0 (−6.0 to 6.6)	Mean difference	0.25 (−1.73 to 2.23)	.80	0.93 (−1.02 to 2.88)	.34
End of study and old EDW	15	3.5 (−2.7 to 8.0)	29	0.0 (−4.6 to 8.0)	Mean difference	2.49 (0.78-4.2)	.005	2.47 (0.63-4.3)	.01
Post-HD 3 months post-study and end of study	15	0.0 (−8.6 to 3.3)	29	0.7 (−11.0 to 3.1)	Mean difference	−0.56 (−2.36 to 1.24)	.53	0.03 (−1.70 to 1.76)	.97
Average pre-HD wks 3-5 and	15	0.2 (−7.7 to 6.0)	29	0.1 (−7.8 to 7.7)	Mean difference	0.14 (−1.59 to 1.87)	.87	0.21 (−1.64 to 2.06)	.82
pre-study									
Average post-HD wks 3-5 and pre-study	15	0.2 (−1.0 to 10.8)	29	0.1 (−2.3 to 7.2)	Mean difference	0.67 (−0.75 to 2.09)	.35	0.94 (−0.53 to 2.42)	.2
Average post-HD wks 3-5 and at 3 months post-study	15	−0.1 (−3.5 to 7.9)	29	−0.1 (−2.0 to 9.2)	Mean difference	−0.03 (−1.68 to 1.62)	.97	−0.66 (−2.10 to 0.78)	.36
Average post-HD wks 3-5 and new EDW	15	1.5 (−2.1 to 6.2)	29	0.5 (−3.2 to 9.0)	Mean difference	0.22 (−1.22 to 1.67)	.76	0.28 (−1.22 to 1.78)	.71
Average post-HD wks 3-5 and original EDW	15	0.5 (−3.1 to 5.2)	29	0.5 (−3.2 to 9.0)	Mean difference	−0.2 (−1.71 to 1.31)	.79	−0.16 (−1.75 to 1.42)	.83
Old EDW and new EDW	15	0.5 (0.5-1.0)	29	0.0 (−2.0 to 5.5)	Mean difference	0.42 (−0.31 to 1.16)	.25	0.44 (−0.34 to 1.23)	.26
Old EDW and post 3 mo target EDW	15	0.0 (−3.0 to 10.0)	29	0.0 (−6.0 to 6.5)	Mean difference	−0.13 (−1.93 to 1.67)	.88	−0.73 (−2.52 to 1.06)	.41
New EDW and post 3 mo target EDW	15	−1.0 (−4.0 to 9.0)	29	0.0 (−6.0 to 6.0)	Mean difference	−0.55 (−2.24 to 1.13)	.51	−1.17 (−2.83 to 0.49)	.16

a EDW, estimated dry weight; HD, hemodialysis.

b Mean differences, 95% CIs, and *P* values result from linear regression models. Mean differences are interpreted as the difference in means of the given outcome between POCUS and non-POCUS patients (ie, POCUS minus non-POCUS). Odds ratios, 95% CIs, and *P values* result from logistic regression models. Odds ratios are interpreted as the multiplicative increase in the odds of the given outcome for POCUS patients were compared with non-POCUS patients.

c Logistic regression analysis was not possible for challenge EDW and hospitalization for hypervolemia due to the presence of a zero cell count; *P values* result from Fisher’s exact test. In multivariable analysis, logistic regression models were adjusted for dialysis vintage, while linear regression models were adjusted for both dialysis vintage and leg/abdomen edema; we could not adjust for both dialysis vintage and leg/abdomen edema in logistic regression analysis owing to the fewer number of variables that can reasonably be included in logistic regression models compared to linear regression models, and therefore we adjusted for dialysis vintage only which had the lower *P*-value when comparing POCUS versus non-POCUS patients.

d Multivariable analysis was not performed for blood pressure medication reduction and falls owing to the rare occurrence of these variables.

In multivariable analysis adjusting for potential confounding variables, we adjusted for both dialysis vintage and leg/abdomen edema in linear regression analysis involving continuous outcomes, and for dialysis vintage only in logistic regression analysis involving categorical outcomes due to the smaller number of variables that can be reasonably adjusted for in logistic regression compared to linear regression.^17^ Results were consistent in this multivariable analysis, where end of study weight minus new EDW (mean difference: 2.91 kg, *P*=.001) and end of study weight minus old EDW (mean difference: 2.47 kg, *P*=.010) were significantly higher in POCUS patients. At 3 months, the difference between post-HD weight at 3 months and new EDW was comparably lower, although not statistically significant (0.93 kg, *P*=.34) (Figure 4).

**Figure 4:**
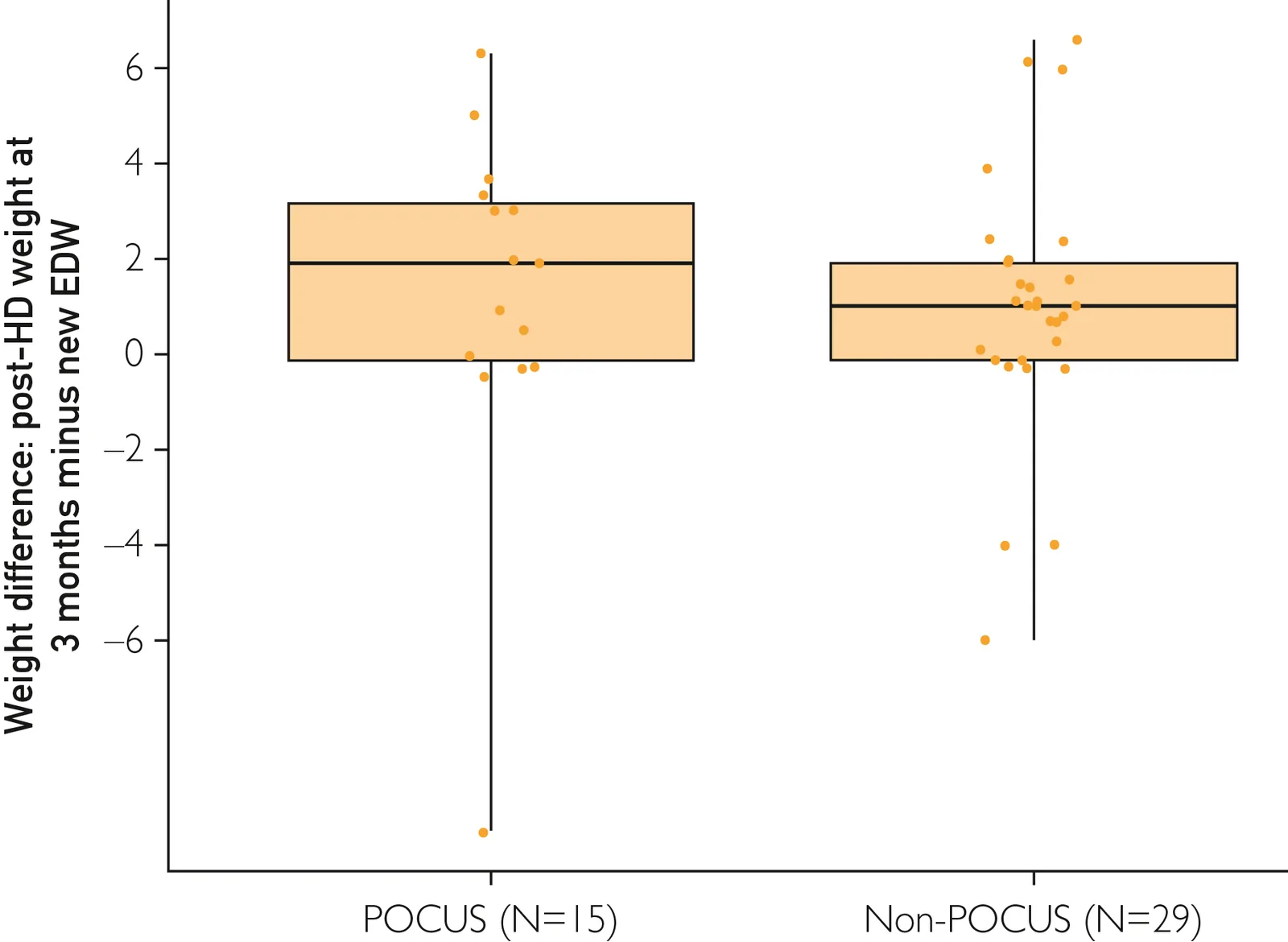
Post-HD weight at 3 months minus new EDW in POCUS and non-POCUS patients. Boxplots show the nonsignificant difference between posthemodialysis weight at 3 months and newly prescribed EDW in the POCUS and non-POCUS groups (mean difference, 0.93 kg; *P*=.34). EDW, estimated dry weight; POCUS, point-of-care ultrasound.

For evaluation of participants meeting prescribed EDW, in the pre-study evaluation, 60% (n=9) of the POCUS group participants were meeting set EDW compared with 72.4% (n=21) of the control group. At the end of the study (weeks 3-5), the proportion of patients meeting EDW decreased for both groups (33.0% POCUS and 58.6% control, OR 0.24 *P*=.059).

No other statistically significant differences in outcomes were identified (all *P*≥.05, Table 2). This included no statistically significant differences between lowest average intradialytic BPs or intradialytic BP changes (difference between pre-HD BP measurement and immediate post-HD BP measurement) between any of the study periods (all *P*≥.24). There was no statistically significant difference between all-cause hospitalizations, hypervolemia-associated hospitalizations, falls, serum albumin, or changes in achieved weight differences at 3 months compared to new EDW (all *P*≥.16). There was an observed overall considerable mean difference in blood pressure from POCUS versus non-POCUS patients (difference of systolic −7.16 mm Hg [*P*=.19] and diastolic −4.48 mm Hg [*P*=.27]).

### Qualitative Assessment of Symptoms

In a qualitative survey of adverse symptoms in the POCUS group, severe muscle cramping (13.33%, n=2), mild fatigue (40%, n=6), mild weakness (20%, n=3), and mild shortness of breath (20%, n=3) were reported as symptoms that worsened after the initial POCUS examination (Supplemental Table 2, available online at http://www.mcpiqojournal.org). None of the reported symptoms interfered with patient participation in the study. 100% of patients reported that the POCUS examination was a positive experience and provided an improved understanding and insight of their volume status.

## Discussion

POCUS as an MHT has improved the practice of medicine and is transforming the medical practice landscape. Our study reported that an abbreviated POCUS evaluation for long-term hemodialysis patients in a busy in-center hemodialysis unit was easy to learn, safe, timely, and improved patient satisfaction and potentially compliance with minimal reported adverse symptoms. The POCUS examination was performed primarily by a nephrology fellow and took approximately 6 minutes, reporting both feasibility in skill competency attainment and applicability for the current fast pace of outpatient dialysis practice. In this study, we reported the practicality of incorporating an abbreviated 6-zone POCUS examination, akin to the Bedside Lung Ultrasonography in Emergency protocol presented by Lichtenstein and Mezière (2008), into a monthly or bi-monthly assessment of a patient on in-center hemodialysis.^14^ Performing POCUS during hemodialysis avoids prolonging patient dialysis center visits and health care burden. Our study also showed that this abbreviated exam allowed earlier health care intervention through adjustment of the EDW.

Fluid and hemodynamic management is critical for ESKD dialysis patients.^7^^,^^18^ The superiority of POCUS for earlier detection of hypervolemia has been supported by multiple studies. Pulmonary congestion can be detected earlier on ultrasound as B lines despite the absence of traditional physical examination findings, such as lung rales or pedal edema, which were detected in only 21% and 10% of patients, respectively.^8^ The POCUS finding of B lines as a sonographic equivalent of Kerley B lines on chest X-ray helps to detect pulmonary congestion or edema, and correlates with left ventricular filling pressure.^8^^,^^19^ Studies have shown this can lead to a change in final diagnosis in up to 27% of patients.^8^^,^^18^ In 2020, a RCT examined POCUS’s ability to reduce time to diagnosis of hypervolemia.^9^ It showed that 79% of patients were found to have clinically relevant findings obtained through POCUS that led to earlier time to appropriate treatment by ∼19 hours. Similarly, our study identified hypervolemia in 100% of POCUS participants compared with 46.7% assessed by traditional physical examination, facilitation earlier intervention through EDW adjustment. In 2023, Al-Saray and Ali^1^ reported comparable findings in an outpatient dialysis population, with POCUS detecting pulmonary edema in up to 60% of patients despite absence of leg edema or abnormal lung examination. Our study’s findings support previous evidence that POCUS complements the health care provider’s diagnostic skillset by improving diagnostic accuracy and reducing time to intervention, while reporting its utility in the outpatient dialysis setting. This earlier detection of clinically silent hypervolemia by POCUS could prevent hospitalizations for hypervolemia or exacerbations of heart failure.^7^ A trial with a larger study population and longer intervention phase may be necessary to detect statistically significant differences (*P*≤.05) in outcomes and achievement of prescribed EDW.

All POCUS participants tolerated EDW adjustment and only a minority of participants described dialysis-associated symptoms, comparable with those reported in routine dialysis patients.^19^ POCUS may also provide indirect benefits including, bringing the provider back to the bedside, increasing provider-patient rapport, and improving patient medical compliance and education by showing real-time evidence of hypervolemia.

In this study, IVC diameter and collapsibility were inconsistent with other findings of hypervolemia. Although 60% of POCUS patients had minimal IVC collapsibility, 100% of patients had an IVC diameter of 2.1 cm or less, which prior studies had suggested correlates with normal or low right atrial pressure.^20^ These contrasting findings support that IVC measurement is not a reliable marker of volume status in our study population. IVC has been shown to be affected by multiple nonvolume parameters and may not be accurate for patients with relevant comorbidities or illnesses, such as right ventricular dysfunction, left-sided heart failure, pulmonary hypertension, tricuspid regurgitation, pericardial effusion, venous compliance, and presence of a tunneled dialysis catheter.^21^ Furthermore, studies have suggested that both longitudinal and transverse views help mitigate off-axis IVC assessment, and the sphericity index from the transverse view may correlate more closely to right atrial pressure than a single-plan diameter. This requires simultaneous 3D echocardiography, however, and its use should be investigated further in this patient population.^22^ POCUS IVC evaluation performed at the end of dialysis may additionally reflect intravascular volume rather than total body volume, and may be impacted by catheter-related shear forces. Finally, IVC measurement is confounded by nonvolume parameters, including pulmonary hypertension, heart failure with preserved ejection fraction (40.0%) or reduced ejection fraction heart failure (13.3%), and limitations from user technical ability. For the 60% of patients with IVC collapsibility, 100% met hypervolemia criteria by ultrasound B-lines. This suggests that postdialysis lung ultrasound assessment may more consistently detect residual volume overload in this cohort. Further studies should evaluate the optimal timing of POCUS assessments relative to hemodialysis treatment and evaluate the implications of these time-sensitive findings on clinical outcomes.

### Weights

Our study reported that POCUS identified hypervolemia criteria that prompted EDW reduction more frequently than non-POCUS patients. However, despite earlier and more frequent decreases in prescribed EDW, POCUS did not result in sustained improvements in post-HD weights. At study end (week 4), the median difference between the post-HD weights to the new prescribed EDW was 4.3 kg in the POCUS group when compared with 0.3 kg in the non-POCUS group (mean difference 2.91 kg, *P*=.001). At study end (4 weeks), there was also no statistically significant improvement in post-HD weights compared with either pre-study weights (*P*=.20). At post-study 3 months after, there was no statistically significant improvement in post-HD weights compared with new EDW (*P*=.34).

The absence of a statistical benefit in achieving a reduction in EDW through POCUS may be explained by the small sample size, interpatient variability, and the lack of precision with patient weights, which may reflect changes separate from volume. For example, the range of weight differences in the POCUS group had greater distribution and variability as compared to the non-POCUS group. A small number of POCUS patients who consistently weighed above their EDW may have influenced the study’s finding that POCUS-guided EDW reduction did not improve hypervolemia (Table 2). This is supported by fewer POCUS patients at baseline meeting the prescribed EDW before the study (60.0% POCUS compared with 72.4% control; *P*=.50). Patient tolerability for volume removal, compliance, changes in medications, patient urine output, non-study dialysis providers’ change in patient management, or other confounders may have also contributed to these findings. The inability to reach the new EDW may have further been due to the intentionally restricted conservative EDW reductions in the study protocol, which was designed to maximize safety and minimize patient harm. The study protocol did not allow for adjustment of dietary or fluid intake recommendations, medications, or dialysis prescriptions, including sodium or ultrafiltration modeling profiles, bicarbonate concentration, dialysate temperature, treatment duration, or intravenous saline administration.^4^^,^^7^^,^^10^^,^^23^ These interventions may facilitate achievement of volume management goals but were beyond the scope of this study. Although POCUS patients did not reach the new EDW at either end of study or 3 months, there were no adverse events (see below) or increased symptom burden.

### Feasibility and Safety

We assessed the feasibility and safety of conducting an abbreviated, time-efficient POCUS examination in a real-world outpatient in-center hemodialysis setting. We targeted determining if this assessment could be reliably conducted within the dialysis unit without increasing patient or provider burden. Our study used an affordable, portable ultrasound device that conducted a simplified POCUS examination using hypervolemia criteria that consisted of either detection of at least 3 confluent ultrasound B lines in more than 2 of 6 lung zones with at least 1 zone positive bilaterally or an IVC diameter of greater than or equal to 2.1 cm with <50% diametric collapsibility. This study design is similar to the LUST trial cutoff for mild congestion of 5 or greater ultrasound B lines^8^ and the 4-zone/3-line protocol of the POCUS in dialysis study from Iraq in 2023.^1^ Previous studies reported efficacy in dialysis patients,^1^^,^^4^^,^^10^^,^^19^^,^^21^^,^^24^ but uptake on use in daily clinical practice is limited due to concerns about feasibility as related to operator training, time, patient logistics and positioning, and patient satisfaction. We report that performing this abbreviated POCUS examination was feasible with rapid operator skill acquisition with an average POCUS assessment time decreasing from 7.2 minutes to 6.1 minutes between the first and second assessment during the study. This time could further decrease with routine practice and could be incorporated as one of the monthly reimbursable visits.

Regarding patient safety, there was no statistical difference for hospitalizations, falls, or intradialytic hypotension between the POCUS and non-POCUS groups. There were more hospitalizations for hypervolemia in the non-POCUS group (2 vs 0), but due to small sample sizes, statistical significance for hypervolemia-associated hospitalizations could not be assessed. The most common symptoms reported were muscle cramping (13.33%), fatigue (40%), and weakness (20%), although none of the patients reporting these symptoms were severe enough to withdraw from the study or alter the new EDW. The prevalence of these symptoms parallels the general dialysis population (Supplemental Table 2).^25^

From a qualitative perspective, study investigators received an overwhelmingly positive patient response with regard to their study experience based on patient surveys. All patients in the POCUS group stated that the POCUS study period was a positive experience and that they felt more involved with clinical decision-making for their care. This was an unexpected finding which may infer that POCUS assessment in clinical practice may positively influence patient engagement and compliance in volume management.

### Blood Pressure Effect and Differences

No statistically significant difference was found between average measured pre-dialysis, postdialysis, or lowest intradialytic BP during the study (all *P*≥.19). This suggests that POCUS-guided EDW intervention did not exacerbate dialysis-related hypotension.

The POCUS group had a marked greater reduction in systolic and diastolic BP (without associated hypotension) between pre-dialysis and postdialysis measurements during weeks 3-5 (*P*=.24 and *P*=.75, respectively), compared with non-POCUS. The systolic BP reduction was 13 mm Hg in POCUS group versus 7 mm Hg in non-POCUS group, and the diastolic BP was 6 mm Hg in POCUS group versus 4 mm Hg in the non-POCUS group. This suggests that BP control may be improved with POCUS-guided interventions on EDW prescription changes. The small number of patients in this study with significant BP variability may not have allowed for statistical significance.

Intradialytic BP change, measured as the difference between immediate pre-HD BP measurement and immediate post-HD BP measurement, was additionally assessed. The variability in intradialytic BP reduction would support the findings that intradialytic BP is an unreliable measurement of volume status changes, and cardiovascular outcomes are better correlated with ambulatory blood pressure monitoring.^26^ A study investigating the correlation between POCUS examination and non-dialysis home BP measurements or a 24-hour ambulatory BP monitor may provide better evidence on the impact of POCUS-guided interventions on BP management.

### Limitations

Several limitations are important to acknowledge. First, the sample size is small, and therefore the possibility of type II error (ie, a false-negative finding) is important to consider. We cannot conclude that a true difference between attained dry weights between POCUS and non-POCUS patients does not exist due to the occurrence of a nonsignificant *P*-value in this study. For instance, comparatively, more POCUS patients had heart failure and were unable to reach their prescribed EDW even before study, potentially limiting the effectiveness of POCUS-guided interventions in achieving sustained EDW. Larger, adequately powered randomized studies are needed in this at risk dialysis population. Second, the study phase period was a short, 4-week interval, and investigators were not blinded. Third, the 6-zone protocol has not been previously validated against the 8-zone protocol in previous studies.^15^ Our study adds to the safety of its use in dialysis patients, but direct comparison studies are needed. Fourth, the use of primarily a single physician as the ultrasound operator for the study was intended to limit inter-operator variability and confounders. This practice reflected the literature review from other nephrology POCUS studies that reported high interobserver reliability and inter-probe concordance in patients on hemodialysis, but the possibility of skewing cannot be completely ruled out.^7.9^ Fifth, patients reported positive experiences and increased knowledge from POCUS examinations, which may improve patient satisfaction and compliance; however, the Hawthorne effect could be a confounding variable on study results. A future study intentionally investigating the impact of POCUS on patient compliance measures and its translation to improved patient outcomes would be impactful. Finally, antihypertensive medications were more frequently reduced in the non-POCUS group. This may be attributed to differences in management of medications, dialysis prescriptions, or ultrafiltration in the non-POCUS group, as this group had a greater range in differences between new EDW and old EDW suggestive of more significant volume removal. Medication use, including diuretics for patients with residual renal function, were not assessed and may have influenced volume status and the ability to achieve prescribed EDW and the outcomes of this study.

### Future Study Opportunities

Further studies with POCUS assessments over a longer study period and more substantial adjustments to EDW may have more significant impact on achieved target weights and clinical outcomes. Consistent with this possibility, the mean difference between postdialysis weight and newly prescribed EDW decreased from 2.91 kg at study completion to 0.93 kg at 3-month follow-up, suggesting that the effects of EDW adjustment may require longer follow-up to become apparent. Prolonged multifaceted interventions with the new prescribed EDW may be required to achieve relevant impact.

Our findings also suggests that limited IVC measurements may have limited utility for assessing hypervolemia in dialysis patients, and further investigation on the use of alternative POCUS techniques (eg, venous excess ultrasound scoring system) or IVC sphericity index may be beneficial for hypervolemia assessment.^27^ This scoring system has been evaluated in the hospital setting, and its use in outpatient dialysis populations at risk for hypervolemia warrants further study.

Finally, the inability to attain EDW or improve exploratory endpoints in our POCUS group likely reflects the complexity and multiple factors impacting the dialysis population. As this was an initial study, we were intentionally conservative on our POCUS-guided interventions to avoid patient harm. We hypothesize that more intensive POCUS-guided challenges in dry weight with simultaneous patient counseling, medication management, and dialysis prescription modifications may be needed to translate detection of subclinical congestion into meaningful clinical benefit.^23^ Future larger randomized studies with multiple trained POCUS practitioners to should evaluate timing of POCUS evaluation (eg, 3rd session of dialysis of the week), the incorporation of holistic patient counseling, medication, and dialysis prescription interventions, and the inclusion of qualitative symptoms for both study groups on how abbreviated POCUS-guided interventions affect hard clinical outcomes, dialysis-associated symptoms, patient satisfaction, and medical compliance.

## Conclusion

In this study, an abbreviated POCUS protocol to guide volume removal in long-term outpatient hemodialysis patients was feasible, efficient, safe, and applicable for diagnosis and management. The modified 6-zone lung protocol is easy to learn with repeated assessment over this short study, reporting improved exam times to an average of 6.1 minutes. Patients favored the POCUS experience in bringing the provider back to bedside, potentially improving patient compliance and satisfaction. Future quality improvement projects may include instruction in POCUS assessment to physicians and health care providers, thereby incorporating its sustained use in monthly assessments and preventing complications of hypervolemia. Larger prospective randomized studies utilizing comprehensive interventions to achieve a meaningful change in EDW based on in-center hemodialysis POCUS assessments over a longer interventional period are needed to further substantiate its utility in everyday practice.

## Potential Competing Interests

The authors report no competing interests.

## Ethics Statement

This study was reviewed and approved by the institutional review board and participants have signed written consent.
