# Supplementary material for: Abbreviated Point-of-Care-Ultrasound Volume Assessment for Outpatient Hemodialysis Patients
Source: Mayo Clin Proc Innov Qual Outcomes. 2026 Oct 9;10(6):100766. doi: 10.1016/j.mayocpiqo.2026.100766 (PMC13677595; doi:10.1016/j.mayocpiqo.2026.100766)
Supplement: Supplementary file 1 [file mmc1.docx]

**Appendix**

| Term | Definition |
| --- | --- |
| Euvolemia | Identified by the absence of at least +1 pitting edema in bilateral shins or pulmonary rales on exam |
| Challenged EDW | If EDW was reduced even one-time for either POCUS or non-POCUS group during any point in the study period |
| Pre-dialysis BP | Measured systolic and diastolic BP immediately pre-dialysis treatment |
| Post-dialysis BP | Measured systolic and diastolic BP immediately post-dialysis treatment, averaged over 6 treatments in each study phase |
| Lowest average intra-dialytic BP | Lowest BP measured during individual hemodialysis treatments, averaged over 6 treatments in each study phase |
| Intradialytic BP | The difference between pre-HD BP measurement and immediate post-HD BP measurement |
| Pre-HD weights | Measured standing weight pre-dialysis treatment averaged over 6 treatments in a study phase |
| Post-HD weights | Measured standing weight post-dialysis treatment averaged over 6 treatments in a study phase |
| Old EDW | Targeted estimated dry weight pre-study |
| New EDW | Targeted estimated dry weight at end of study week 4 |
| End of study weight | Measured standing weight post-dialysis treatment at end of study week 4 |
| Post-HD weight at 3 months | Measured standing weight post-dialysis treatment at end of 3-month observation phase post-study intervention |
| Mean difference (MD) | Calculated mathematical difference between two measured inputs |

**Appendix Table 1:** Definitions

**Appendix Table 2.** Patient Survey Results and changes in symptoms between beginning and end of study for POCUS patients.

| **Severity** | **Muscle Cramping** | **Fatigue** | **Shortness of Breath** | **Nausea** | **Weakness** | **Dizziness** |
| --- | --- | --- | --- | --- | --- | --- |
| Severe | 13.33% (2/15) | -6.67% (1/15) | -13.33% (2/15) | 0.00% (0/15) | 0.00% (0/15) | 0.00% (0/15) |
| Moderate | 0.00% (0/15) | -33.33% (5/15) | -6.67% (1/15) | 0.00% (0/15) | -20.00% (3/15) | 0.00% (0/15) |
| Minimal | -13.33% (2/15) | 40.00% (6/15) | 20.00% (3/15) | 0.00% (0/15) | 20.00% (3/15) | 0.00% (0/15) |

Positive = increased symptom reporting. Negative = decreased symptom reporting.

**Appendix Figure 1: Lung POCUS 6-zone protocol (right chest).**

Three zones assessed by POCUS bilaterally: mid-clavicular (1), mid-axillary(2), and posterior axillary line (3). Image shows zones on right and were also measured in mirror image on the left and denoted L1, L2, L3.

R1

R2

R3

**Appendix Figure 2: Sample patient survey questionnaire**

4-Week Patient Questionnaire

Patient During the past 4 weeks do you recall feeling the following:

| Symptom | More than half the days per week | Less than half the days per week | Less than 1 day per week |
| --- | --- | --- | --- |
| Muscle cramping |  |  |  |
| Fatigue |  |  |  |
| Shortness of Breath |  |  |  |
| Nausea |  |  |  |
| Weakness |  |  |  |
| Dizziness |  |  |  |

Do you feel that your symptoms have worsened since start of the study?

Yes	No

Did you have other symptoms not listed here? List below:

Please list other comments about your study experience:

**Appendix Figure 3:** Hypervolemia assessment by POCUS versus physical exam in the POCUS group at week 1 and week 3.

Appendix Figure 3:Comparing hypervolemia assessment by POCUS b-lines, physical exam and IVC collapsibility.

Blue indicates POCUS Week 1. Orange indicates POCUS Week 3.

POCUS >3 B LINES

PHYSICAL EXAM

IVC COLLAPSIBILITY
